# Dataset on the organic acids, sulphate, total nitrogen and total chlorophyll contents of two lettuce cultivars grown hydroponically using nutrient solutions of variable macrocation ratios

**DOI:** 10.1016/j.dib.2020.105135

**Published:** 2020-01-14

**Authors:** Christophe El-Nakhel, Antonio Pannico, Marios C. Kyriacou, Spyridon A. Petropoulos, Maria Giordano, Giuseppe Colla, Antonio Dario Troise, Paola Vitaglione, Stefania De Pascale, Youssef Rouphael

**Affiliations:** a Department of Agricultural Sciences, University of Naples Federico II, Portici, Italy; b Department of Vegetable Crops, Agricultural Research Institute, Nicosia, Cyprus; c Department of Agriculture, Crop Production and Rural Environment, University of Thessaly, Volos, Greece; d Department of Agriculture and Forest Sciences, University of Tuscia, Viterbo, Italy

**Keywords:** *Lactuca sativa*.L, Malate, Tartrate, Oxalate, Citrate, Isocitrate, NFT, Growth chamber

## Abstract

The data presented in this article were derived from dry and fresh samples of soilless-grown butterhead lettuce (*Lactuca sativa* L. var. *Capitata*). Organic acids, total nitrogen, sulphate and total chlorophyll concentrations varied in response to cultivar (red or green butterhead Salanova) and to nutrient solution macrocation ratios (high proportion of K, Ca or Mg). Kjeldahl, spectrophotometry and ion chromatography were the principal employed methods. Data of total nitrogen and sulphate concentrations contribute to the understanding of macrocation uptake by plants and may drive prospective relevant research. Organic acids are indicators of plant tolerance to stress, including nutrient deficiencies, and the variability of their concentrations provide insights to plant stress physiology. The data reported in this paper are related to the research article “The bioactive profile of lettuce produced in a closed soilless system as configured by combinatorial effects of genotype and macrocation supply composition”, authored by El-Nakhel et al. (2020) [1].

**Table:** Specifications table

Subject	Agricultural and Biological Sciences
Specific subject area	Horticultural Science, Hydroponics, Plant Nutrition
Type of data	Raw data and Table
How data were acquired	ICS-3000 Ion chromatography system, Dionex, Sunnyvale, CA, USA. DR 2000 Spectrophotometer, Hach Co., Loveland, CO, USA. Heating plate and steam distiller, VELP Scientifica, Usmate Velate, MB, Italy
Data format	Raw and Analyzed data
Parameters for data collection	Wiley Mill with a 841 μm screen Heating plate: 420 °C for 30 min Distiller equipped with sodium hydroxide 32% Shaking water bath: 80 °C for 10 min Centrifuge 10 min at 6000 rpm 0.20 μm filter paper IonPac ATC-HC anion trap (9 × 75 mm) AS11-HC analytical column (4 × 250 mm) AG11-HC precolumn (4 × 50 mm) Self-regenerating suppressor AERS500 (4 mm) Spectrophotometer at 647 and 664 nm wavelengths
Description of data collection	The data on leaf total N were acquired by means of digestion, distillation and titration according to the Kjeldahl method [2]. The data on organic acids and sulphate concentrations were obtained by ion chromatography according to Rouphael et al. [3]. The data on total chlorophyll concentration were obtained by spectrophotometry according to Lichtenthaler and Buschmann [4].
Data source location	Institution: Experimental farm of University of Naples Federico II. City/Town/Region: Bellizzi, Salerno Country: Italy
Data accessibility	With the article Raw data (Supplementary material) Mean and standard error of the experimental data are available in this article in Table 1.
Related research article	The bioactive profile of lettuce produced in a closed soilless system as configured by combinatorial effects of genotype and macrocation supply composition. Food Chemistry https://doi.org/10.1016/j.foodchem.2019.125713 [1]

**Table:** 

**Value of the Data** • These data provide additional information regarding lettuce grown under Nutrient Film Technique (NFT) using nutrient solutions of different cationic proportions (K/Ca/Mg) in respect to: (i) how much nitrogen and sulphate was allocated to the plants, and (ii) how total chlorophyll and organic acids contents varied between the different treatments. • These data are useful for vegetable growers in general and plant factories in particular by providing additional options on how to manage the nutrient solution to improve the quality of the final product. They are as well useful to scientists interested in plant cultivation under closed environment systems. • Data of total nitrogen and sulphate concentrations contribute to the understanding of macrocation uptake by plants and may drive prospective relevant research. Organic acids may serve as indicators of plant tolerance to stressors, including nutrient deficiencies, and data describing the variability of their concentrations provide insight to plant stress physiology. • These data might be useful in the nutritional optimization of dietary intake, especially when organic acids constitute a serious consideration (e.g. oxalic acid and kidney failure).

## 1 Data

Accurate application of chemical eustress such as macronutrient deprivation, mild NaCl and constant exposure of roots to different cationic and anionic proportions may modulate the nutritional and functional quality traits in leafy vegetables, including lettuce [[5], [6], [7], [8]]. The dataset contains raw data obtained by Kjeldahl, spectrophotometry and ion chromatography methods from dry and fresh samples of soilless-grown butterhead lettuce (Supplementary material). Information about organic acids (malate, tartrate, oxalate, citrate and isocitrate), total nitrogen, sulphate and total chlorophyll concentrations as a function of butterhead lettuce cultivars and nutrient solution macrocation ratios are displayed in Table 1.

**Table 1:** Total nitrogen, sulphate, organic acids and chlorophyll contents in two butterhead lettuce cultivars grown hydroponically using different nutrient solution macrocation ratios. All data are expressed as mean ± standard error, *n* = 3.

Source of variance	Total N (g kg^−1^ dw)	Sulphate (g kg^−1^ dw)	Malate (g kg^−1^ dw)	Tartrate (g kg^−1^ dw)	Oxalate (g kg^−1^ dw)	Citrate (g kg^−1^dw)	Isocitrate (g kg^−1^dw)	Total chlorophyll (mg kg^−1^ fw)
Cultivar (C)								
Green Salanova	42.35 ± 0.99 b	1.24 ± 0.09 b	41.90 ± 2.35 b	3.04 ± 0.09 a	1.44 ± 0.07 b	12.62 ± 0.24 a	0.55 ± 0.03 a	141.0 ± 3.9 b
Red Salanova	44.92 ± 0.59 a	1.89 ± 0.10 a	44.83 ± 3.03 a	2.22 ± 0.05 b	1.85 ± 0.08 a	9.44 ± 0.77 b	0.37 ± 0.03 b	265.9 ± 8.5 a
Nutrient solution (S)								
S_K_	46.01 ± 0.24 a	1.92 ± 0.16 a	53.05 ± 1.55 a	2.54 ± 0.15 b	1.59 ± 0.10 b	11.81 ± 0.71 a	0.46 ± 0.05	190.0 ± 20 c
S_Ca_	42.71 ± 0.21 b	1.44 ± 0.17 b	38.56 ± 1.41 b	2.47 ± 0.17 b	1.43 ± 0.08 c	9.55 ± 1.35 b	0.45 ± 0.07	202.9 ± 33 b
S_Mg_	42.19 ± 1.54 b	1.33 ± 0.12 b	38.49 ± 2.04 b	2.89 ± 0.24 a	1.92 ± 0.10 a	11.73 ± 0.32 a	0.47 ± 0.01	217.4 ± 32 a
C × S								
Green Salanova × S_K_	45.57 ± 0.09 b	1.58 ± 0.07	50.18 ± 0.61 b	2.87 ± 0.04 b	1.37 ± 0.04	13.22 ± 0.27 a	0.58 ± 0.02 a	146.7 ± 4.7 c
Green Salanova × S_Ca_	42.73 ± 0.29 c	1.06 ± 0.01	41.35 ± 1.26 c	2.84 ± 0.03 b	1.26 ± 0.03	12.51 ± 0.35 ab	0.61 ± 0.03 a	130.3 ± 8.1 d
Green Salanova × S_Mg_	38.76 ± 0.24 d	1.08 ± 0.07	34.17 ± 0.48 d	3.41 ± 0.05 a	1.69 ± 0.02	12.13 ± 0.47 ab	0.45 ± 0.02 b	146.0 ± 2.8 c
Red Salanova × S_K_	46.45 ± 0.27 a	2.26 ± 0.10	55.92 ± 1.82 a	2.21 ± 0.04 d	1.82 ± 0.04	10.41 ± 0.68 c	0.35 ± 0.01 c	233.4 ± 4.7 b
Red Salanova × S_Ca_	42.70 ± 0.35 c	1.82 ± 0.01	35.76 ± 0.76 d	2.09 ± 0.04 d	1.60 ± 0.02	6.59 ± 0.41 d	0.29 ± 0.00 d	275.6 ± 3.5 a
Red Salanova × S_Mg_	45.62 ± 0.29 b	1.59 ± 0.03	42.82 ± 1.35 c	2.36 ± 0.07 c	2.14 ± 0.04	11.33 ± 0.35 bc	0.48 ± 0.01 b	288.8 ± 1.0 a
Significance								
Cultivar (C)	***	***	*	***	***	***	***	***
Nutrient solution (S)	***	***	***	***	***	***	NS	***
C × S	***	NS	***	**	NS	***	***	***

S_K_, S_Ca_, S_Mg_, nutrient solution with high proportion of K, Ca, and Mg, respectively.

ns,*,**, *** Non-significant or significant at *P* ≤ 0.05, 0.01, and 0.001, respectively. Different letters within each column indicate significant differences according to Duncan's multiple-range test (*P* = 0.05).

## 2 Experimental design, materials and methods

### 2.1 Plant cultivation and experimental treatments

The experiment was conducted in an open-gas-exchange climate chamber (28 m^2^) at the experimental farm of the University of Naples Federico II, located at Bellizi, Italy. Red and green pigmented butterhead lettuce cultivars (*Lactuca sativa* L. *var. capitata*) red Salanova® and green Salanova® (Rijk Zwaan, Der Lier, The Netherlands), were chosen for this 19-day experiment, where 3 nutrient solutions having different cationic ratios (high proportion of K, Ca or Mg) were used. Each treatment was replicated three times, placed in a randomized complete-block design [1].

### 2.2 Total N, sulphate and organic acids analysis

Leaf plant tissues were dried at 70 °C for 72 h (until reaching constant weight), and then ground in a Wiley Mill (841 μm screen) before being used for total nitrogen, sulphate and organic acids quantification. Total nitrogen concentration was assessed by the Kjeldahl method [2] in 3 steps: digestion of the dried leaf material, distillation and titration. One g of each replicate was placed in a glass tube where 7 mL of sulfuric acid 96%, 5 mL of hydrogen peroxide 30% and 7 g of catalyzer (selenium + potassium sulphate + copper oxide) were added, then the tubes (La Pyrex®) were placed for digestion in a heating plate DK 20 (Velp scientifica, Usmate Velate, MB, Italy) for 30 min at 420 °C. The distillation was carried out through a UDK 140 distiller (Velp scientifica, Usmate Velate, MB, Italy) with the addition of 50 mL of 32% sodium hydroxide. Finally, the titration was conducted with sulfuric acid 0.1 N in the presence of a colored indicator (methyl red + Bromocresol green) and the titration volume was used to calculate total nitrogen percentage which was converted to g of nitrogen per kg of dry weight.

For organic acids and sulphate, 250 mg of the dry material were suspended in 50 mL of ultrapure water (Milli-Q, Merck Millipore, Darmstadt, Germany) and then shaken in a water bath at 80 °C for 10 min (ShakeTemp SW22, Julabo, Seelbach, Germany). The samples were placed in a centrifuge for 10 min at 6000 rpm (R-10 M, Remi Elektrotechnik Limited, India), then filtered through a 0.20 μm filter paper (Whatman International Ltd., Maidstone, U.K.) The tested elements were separated by ion chromatography (ICS-3000, Dionex, Sunnyvale, CA, USA) and quantified through ion chromatography coupled to a conductivity detector. An IonPac ATC-HC anion trap (9 × 75 mm), and an AS11-HC analytical column (4 × 250 mm) equipped with an AG11-HC precolumn (4 × 50 mm) and a self-regenerating suppressor AERS500 (4 mm) were used for the separation. The concentrations were determined against standards of anions ranging from 0.05 to 0.5 mM and were expressed as g kg^−1^ dry weight, as previously described by Rouphael and co-workers [3].

### 2.3 Total chlorophyll analysis

For the photosynthetic chlorophyll extraction, 500 mg of frozen fresh leaves were extracted in 3 mL of 90% ammoniacal acetone using a mortar and a pestle, then the final mixture volume was brought to 10 mL with the same solvent and placed in darkness for 15 min, then centrifuged at 3000 g for 3 min. Chlorophyll content was determined by measuring the absorbance of the supernatant at 647 and 664 nm by means of a DR 2000 spectrophotometer (Hach Co., Loveland, Colorado, USA), where ammoniacal acetone 90% was used as a blank. Total chlorophyll was calculated as the sum of chlorophyll a and b by using the formulae and extinction coefficients described by Lichtenthaler and Buschmann [4], and finally expressed as mg kg^−1^ fresh weight.

### 2.4 Experimental data analysis

Experimental data were subjected to two-way analysis of variance (ANOVA) using the software package SPSS 13 (SPSS Inc., Chicago, Ill.). The Duncan's Multiple Range Test was performed at P ≤ 0.05, in order to separate treatment means within each measured parameter.

## References

[1] El-Nakhel C., Petropoulos S.A., Pannico A., Kyriacou M.C., Giordano M., Colla G., Troise A.D., Vitaglione P., De Pascale S., Rouphael Y. (2020). The bioactive profile of lettuce produced in a closed soilless system as configured by combinatorial effects of genotype and macrocation supply composition. Food Chem..

[2] Bremner J.M., Black C.A., Evans D.D., White I.L., Ensminger L.E., Clark F.E. (1965). Total nitrogen. Methods of Soil Analysis.

[3] Rouphael Y., Colla G., Giordano M., El-Nakhel C., Kyriacou M.C., De Pascale S. (2017). Foliar applications of a legume-derived protein hydrolysate elicit dose-dependent increases of growth, leaf mineral composition, yield and fruit quality in two greenhouse tomato cultivars. Sci. Hortic..

[4] Lichtenthaler H.K., Buschmann C. (2001). Chlorophylls and carote-noids–measurement and characterisation by UV-VIS.–Current Protocols in Food Analytical Chemistry (CPFA), (Supplement 1), F4.3.1–F4.3.8.

[5] Kyriacou M., Rouphael Y. (2018). Towards a new definition of quality for fresh fruits and vegetables. Sci. Hortic..

[6] Rouphael Y., Kyriacou M.C., Petropoulos S.A., De Pascale S., Colla G. (2018). Improving vegetable quality in controlled environments. Sci. Hortic..

[7] Rouphael Y., Petropoulos S.A., Cardarelli M., Colla G. (2018). Salinity as eustressor for enhancing quality of vegetables. Sci. Hortic..

[8] El-Nakhel C., Pannico A., Kyriacou M.C., Giordano M., De Pascale S., Rouphael Y. (2019). Macronutrient deprivation eustress elicits differential secondary metabolites in red and green-pigmented butterhead lettuce grown in a closed soilless system. J. Sci. Food Agric..

